# Valley-selective directional emission from a transition-metal dichalcogenide monolayer mediated by a plasmonic nanoantenna

**DOI:** 10.3762/bjnano.9.71

**Published:** 2018-03-02

**Authors:** Haitao Chen, Mingkai Liu, Lei Xu, Dragomir N Neshev

**Affiliations:** 1 Nonlinear Physics Centre, Research School of Physics and Engineering, Australian National University, Canberra, ACT 2601, Australia; 2 College of Advanced Interdisciplinary Studies, National University of Defense Technology, Changsha 410073, China

**Keywords:** 2D materials, multipolar emission, nanoantenna, plasmonic, transition-metal dichalcogenides, valley polarization

## Abstract

**Background:** Two-dimensional (2D) transition-metal dichalcogenides (TMDCs) with intrinsically crystal inversion-symmetry breaking have shown many advanced optical properties. In particular, the valley polarization in 2D TMDCs that can be addressed optically has inspired new physical phenomena and great potential applications in valleytronics.

**Results:** Here, we propose a TMDC–nanoantenna system that could effectively enhance and direct emission from the two valleys in TMDCs into diametrically opposite directions. By mimicking the emission from each valley of the monolayer of WSe_2_ as a chiral point-dipole emitter, we demonstrate numerically that the emission from different valleys is directed into opposite directions when coupling to a double-bar plasmonic nanoantenna. The directionality derives from the interference between the dipole and quadrupole modes excited in the two bars, respectively. Thus, we could tune the emission direction from the proposed TMDC–nanoantenna system by tuning the pumping without changing the antenna structure. Furthermore, we discuss the general principles and the opportunities to improve the average performance of the nanoantenna structure.

**Conclusion:** The scheme we propose here can potentially serve as an important component for valley-based applications, such as non-volatile information storage and processing.

## Introduction

The inversion-symmetry breaking and quantum confinement in monolayer TMDCs offer unprecedented opportunities to explore valley-based physics and applications [1–3]. The valley pseudospin is associated with the degenerate energy extrema in momentum space [3]. In monolayer TMDCs that have a hexagonal lattice structure valleys of degenerate energy locate at the corners of the hexagonal Brillouin zone: the K and K′ points [4–5]. In analogy to spintronics, the valley pseudospin could also be used as non-volatile information storage and processing, which is known as valleytronics [6–10]. In particular, monolayer TMDCs with direct bandgap at the K and K′ points [11] make it possible to control the valley degree freedom entirely optically. Optical pumping of excitons of a specific valley polarization has been demonstrated by polarization-resolved photoluminescence (PL) measurements [12–14], where the chirality of the PL emission is the same as the pumping light, since different valleys are addressed by the angular momentum of the excitation. Hence, one can switch the chirality of the PL emission from left to the right (and the other way around) by changing the polarization states of the pump beam. It can be envisioned that the dynamic excitation and control of carriers in different valleys is crucial for future valley-based information technologies and applications.

Inspired by such opportunities, a valley-based light emitting diode with controllable emission polarization [15], valley Hall effect [16], and valley-dependent photogalvanic effect have been explored. Excitonic valley coherence [17], valley- and spin-polarized Landau levels [18] and valley Zeeman effect [19–22] have also been studied in monolayer TMDCs. Different schemes to control the valley pseudospin in 2D TMDCs have been developed, including optical [23–24], magnetic [25–26] and electrical [17,27] control.

On the other hand, to facilitate device integration, it is preferable that light emission from 2D TMDCs can be controlled at the nanoscale. Recent advances in resonant metallic nanostructures, referred to as plasmonic nanoantenna, have shown great flexibility and capability for manipulation of the radiation of closely placed emitters [1,3,28]. Plasmonic nanoantenna could significantly modify the emission rate, the radiation pattern and the polarization of emission when their plasmonic modes are excited [29–31]. In particular, localized emitters could effectively excite the higher-order modes of the nanoantenna, which are usually only weakly coupled to free-space plane waves [32–33] but can dramatically modify the radiation of the emitters. Importantly, the near-field and far-field interference of the multiple plasmonic modes present in the nanoantenna offer an unprecedented capability to control all aspects of the emission of localized emitters [33–35]. While the radiation enhancement of emitters by multipolar antennas has been widely studied [33,36–37], the control of the directionality of emission is less explored. Although previous studies [38–42] have shown several designs for spin-dependent directional emission, these schemes are extremely sensitive to the position of the emitter to the nanoantenna, e.g., the directionality of emission would be reversed if the emitter is placed on the opposite side of the antenna. As such, the currently proposed schemes can not be employed to control the emission of delocalized chiral emitters, such as emission from exciton from the two valleys of a 2D TMDC material.

Here, we propose a plasmonic TMDC–nanoantenna system that can effectively route light emission from different valleys of TMDCs into opposite directions. Our nanoantenna can support electric dipole and electric quadrupole resonances, which can be excited, with engineered phases and amplitudes, by the chiral point-dipole emitters corresponding to each valley. Based on the phase-locked excitation and interferences of these resonances, we have shown that the scattering direction of the TMDC–nanoantenna system is valley-locked. Since the different valleys in TMDC can be addressed optically by circularly polarized optical pumping, we then can tune the emission direction of this coupled system by simply changing the circular polarization states of the pumping light. We believe that our scheme could provide useful insight for design of novel component such as couplers and routers in future valley-based information processing devices.

## Results and Discussion

### Concept

At resonances, the far-field radiation of the nanoantennna could be expanded into multipolar series. Equation 1 shows the first three terms, including the contribution of the electric dipole **p**, the electric quadrupole 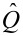 and the magnetic dipole **m** [43]:

[1]
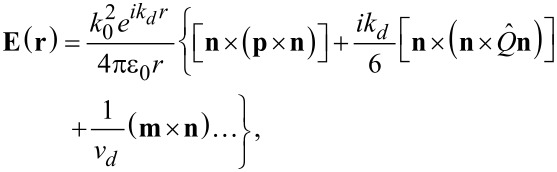


where *k*_0_ is the vacuum wavenumber and *k_d_* is the wavenumber in the surrounding medium. *v_d_* is the speed of light in the medium, **n** is the unit vector in the direction of emission, **r** is the coordinate vector, r = |**r**|.

From Equation 1, we can see that the far-field radiation is in-phase with the electric dipole moment, while there is a π/2 phase difference for the electric quadrupole. Thus, there is naturally a π/2 relative phase difference between the electric dipole and quadrupole contribution to the far-field emission, when their corresponding charges oscillate in phase. In this case, the parallel electric field components of electric dipole and electric quadrupole emission will interfere with each other depending on their relative phase and amplitude. When the amplitudes of the far-field components are comparable, the interference will be constructive in one direction and destructive in the other, for π/2 or 3π/2 phase difference between the electric dipole and quadrupole. On the other hand, the far-field interference is prevented if the phase difference is 0 or π. Our design is based on this interference property, to tailor the emission directions from different valleys.

**Figure 1:**
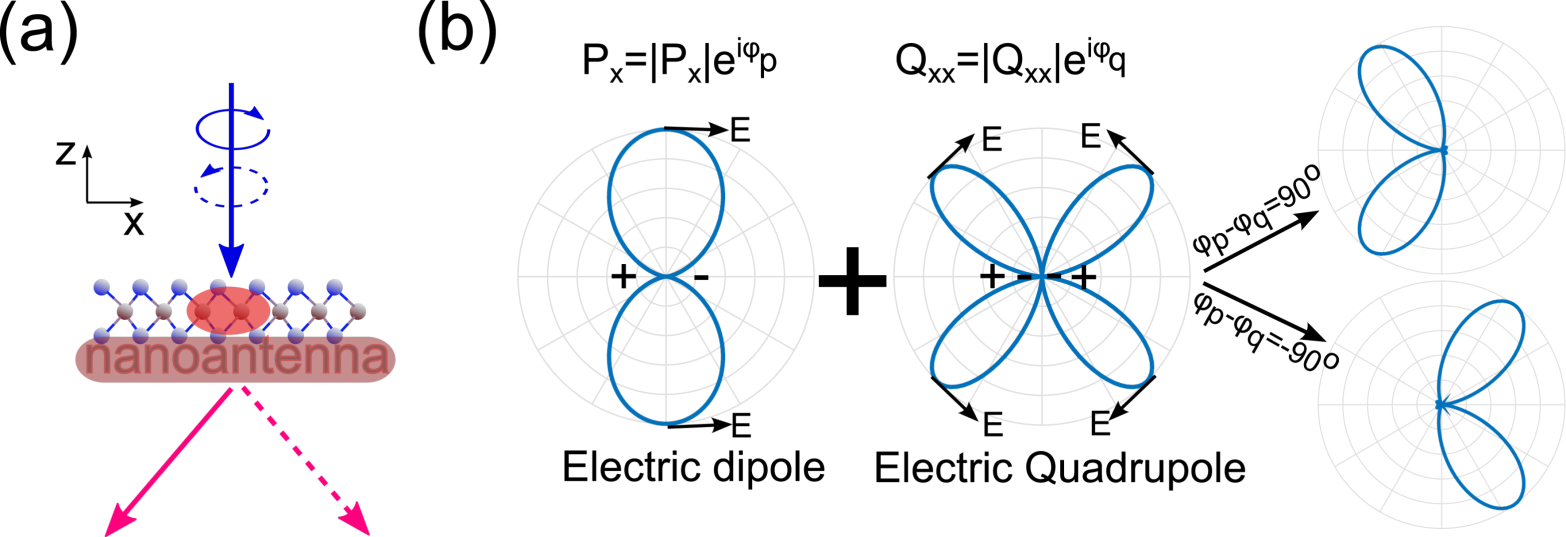
Design principles of the directional emission from the different TMDC valleys. (a) The proposed scheme to separate emission from different valleys through integrating with a properly designed nanoantenna. The directions of the emission from monolayer TMDCs depend on the polarization states of the excitation when different valleys are excited. (b) The working principle of the nanoantenna is based on the interference between the electric fields radiated from an electric dipole and an electric quadrupole; the direction of constructive interference depends on their relative phase.

The basic idea of our concept for splitting the emission from the two valleys of the TMDCs via near-field coupling to a plasmonic nanoantenna is schematically shown in Figure 1a. When the coupled TMDC–nanoantenna system is excited by light of different circular polarizations, the emission from the two valleys (K and K′) will be emitted into opposite directions, as depicted by the two red arrows. The general concept of such valley splitting relies on the interference of multipolar modes excited in the nanoantenna, namely an electric dipole and an electric quadrupole, as shown in Figure 1b. When the parallel electric dipole and quadrupole are excited simultaneously with comparable amplitudes, the radiation direction will depend on the phase difference between the dipole and quadrupole, as discussed above. Thus, by changing the relative phase between the dipole and quadrupole from +π/2 to −π/2, we could effectively tune the radiation direction from one (solid arrow) to the other (dashed arrow).

### Design and implementation

Since the emission from the TMDC monolayer solely comes from the in-plane exciton or trion charge carriers [44], the TMDC emitters could be practically modeled as chiral point-dipole emitters placed in the vicinity of the photonic structure [45]. To emulate such chiral emitters, corresponding to the two valleys in the TMDC monolayer, in our simulations we use two in-plane but orthogonal point dipoles that oscillate with π*/2* phase difference (to emulate the rotation of the dipole). When a +π/2 or −π/2 phase difference is applied between these two emitters, we can emulate left or right chiral emitters [46] from carriers in the K and K′ valleys [47].

**Figure 2:**
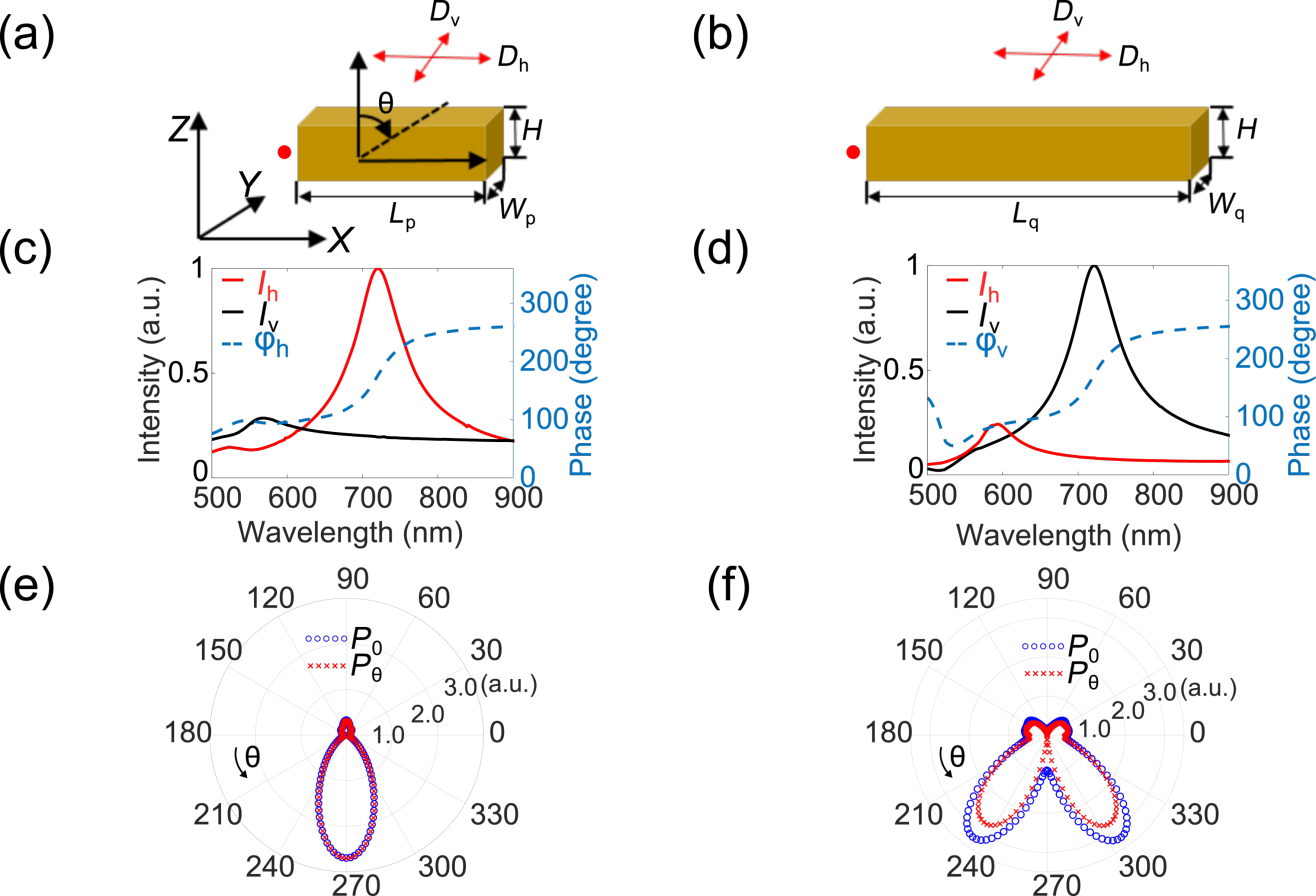
Characteristics of the bar antenna excited by an electric dipole emitter. (a, b) Schematic of the simulation setup for the short bar (a) and the long bar (b) antenna, respectively. The red arrows represent point-dipole emitters with *X* or *Y* orientations. The red spots represent electric probes. (c, d) The electric field intensity (*I*) and phase (φ) at the position of the probe when point-dipole emitters oriented along *X* and *Y* are used as an excitation source for the short and long bar, respectively. The subscript h and v represents the field induced by the point-dipole emitter along *X* and *Y* directions, respectively. We only show phase information for the short bar excited by a *X*-orientated emitter (φ_h_) and the long bar excited by a *Y*-oriented emitter (φ_v_). The field intensity is normalized to the larger one for emitters with different orientations. (e, f) Polar plot of the total (*P*_0_) and azimuthal components (*P*_θ_) of the far-field power distribution when the short bar is excited by a *X*-oriented dipole (e) and the long bar is excited by a *Y*-oriented dipole (f), respectively. The direction of θ is illustrated by arrows.

To achieve such a functionality, we need to construct an antenna that supports both an electric dipole and an electric quadrupole of the same strength and at the same operation wavelength. We hence start with a simple plasmonic nanoantenna consisting of two gold bars of different length. Different plasmonic modes could be excited in such a double-bar antenna when a localized emitter is placed in its proximity. The excitation of plasmonic modes depends on a couple of factors, including the antenna size, as well as the emitter position and orientation. By choosing proper size parameters, either the electric dipole mode or the “dark” quadrupole mode of the antenna can be excited predominantly by the point-dipole source. We choose a short bar with length *L*_p_ = 104 nm and width *W*_p_ = 25 nm, and a long bar with length *L*_q_ = 310 nm and width *W*_q_ = 70 nm. The height of the two bars is the same, 40 nm. A view of each bar of the antenna is shown in Figure 2a and Figure 2b, respectively. Two electric dipoles oriented along *X* (*D*_h_) and *Y* (*D*_v_) are placed 5 nm above the antenna in *Z* direction. The point-dipole emitters are 25 nm away from the bar antenna in *Y* direction. To mimic a practical experimental arrangement, the antenna is placed on top of a glass substrate. Electrical probes are located at the end of each gold bars to detect the electric phase and amplitude.

We start by studying the individual responses of each antenna bar, when excited by a local dipole emitter, orientated parallel or perpendicular to the bar. We perform numerical calculations using finite-integral frequency-domain simulations (CST Microwave Studio) with open boundary conditions. To avoid unphysical sharp edges, we model the gold bars having rounded corners with a radius of curvature of 5 nm. The permittivity of the gold in the visible and near-infrared spectral region is modeled based on experimental data from [48].

The intensity of the electric field along *X* direction (*I*) and phase information (φ) at the probe position when the structure is excited by local point-dipole emitters of different orientations, for short bar and long bar, are shown in Figure 2c and Figure 2d, respectively. For better comparison, we normalize the field intensity to the stronger one. As seen, for the short bar (Figure 2c), the field induced by the *X*-oriented point-dipole (*I*_h_) dominates in the wavelength range of 700–750 nm. In contrast, the excited field from the *Y*-oriented point-dipole (*I*_v_) dominates for the long bar in the same wavelength range (Figure 2d). Importantly, both of the electric field intensities show a resonant peak at the same wavelength of 715 nm.

The phase information detected by the probes corresponds to the phases of the oscillating charges, which defines the phases of the dipole and quadrupole moments. The phase information under the dominant excitation, labeled as φ_h_ and φ_v_ are shown in Figure 2c,d with dashed lines. We could observe that the two resonant modes are in phase. To further investigate the nature of the excited modes in each gold bar, we monitor the far-field radiation pattern of the *X*-oriented emitter coupled to the short bar and the *Y*-oriented emitter coupled to the long bar. The radiation patterns at the resonant wavelength are shown in Figure 2e and Figure 2f, respectively. Due to the existence of the high-index substrate, most of the emitted power goes into the lower half space. In the case of a *X*-oriented emitter and a short-bar antenna (Figure 2e), the radiation pattern shows a typical dipole-emission profile. In the case of a *Y*-oriented emitter and a long-bar antenna, the emission shows a typical quadrupole profile (Figure 2f). In the same polar plots, we show both the total radiated power (*P*_0_ – blue circles) and the azimuthal power component (power contributed from azimuthal electric field, *P*_θ_ – red circles). As seen from Figure 2e and Figure 2f, in both cases the azimuthal component (*P*_θ_) is the dominant polarization component, which is expected for both dipole and quadrupole radiation from bar antennas along the *X* direction. By examining the vectorial near-field profiles (shown in Figure S1, Supporting Information File 1), we further confirm that the electric dipole mode is excited dominantly by the *X*-oriented point-dipole, while the electric quadrupole mode in the long bar is excited by the *Y*-oriented point dipole. Thus, the far-field radiation of these dipole and quadrupole will have a phase difference of π/2. Note that we have fine-tuned the geometry of the antennas in order to have them resonate around 715 nm, which matches the experimentally measured emission wavelength of monolayer WSe_2_. The parameters of the two bars are also optimized such that the radiated electric far-fields have comparable intensities.

**Figure 3:**
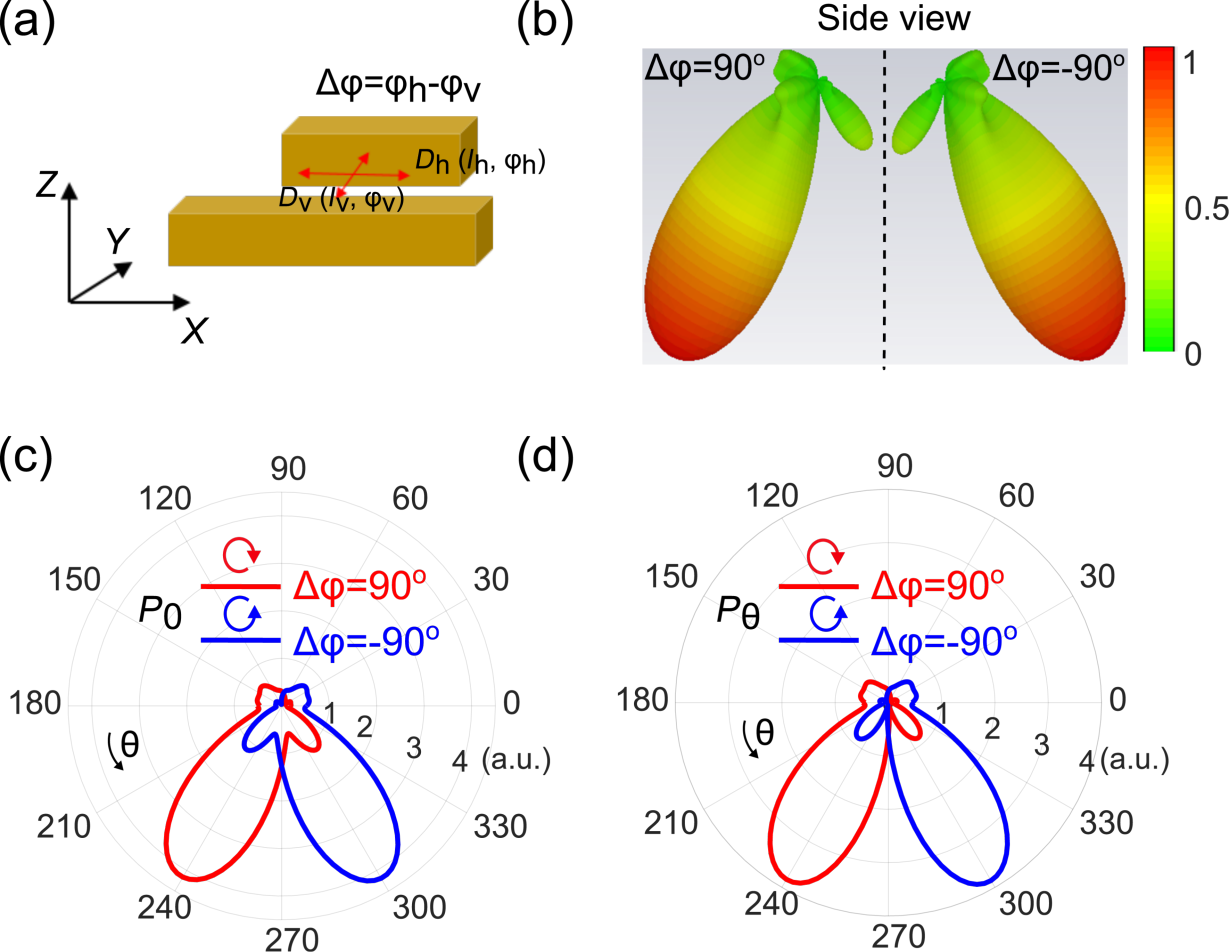
Radiation properties of a double-bar plasmonic antenna. (a) Schematic view of the double-bar antenna. Δφ represents the phase difference between the *X*- and *Y*-oriented point-dipole emitters. Δφ = ±90°is used to mimic right or left chiral emitters, respectively. (b) Side view of far-field pattern when Δφ = ±90°. (c, d) Polar plots of the total radiated power (*P*_0_) and the azimuthal component (*P*_θ_) in the far field, respectively. We show both cases of Δφ = ±90°.

After investigating the response of the individual gold bars, we perform simulation for the combined antenna consisting of two closely spaced gold bars coupled to a chiral point-dipole emitter. A schematic of the antenna–emitter system is shown in Figure 3a. The gap between the two bars is set to 50 nm and the chiral emitter is located in the center of the gap. The chiral emitter is again modeled as two orthogonal electric dipoles with a relative phase of Δφ, where Δφ = ±90°, corresponding to right or left circularly polarized emission, respectively. The calculated radiation patterns of the total emission (side view) are shown in Figure 3b, for both Δφ = 90° and −90°. Due to the interference of the fields emitted from the electric dipole of the antenna and electric quadrupole contributions, the radiation from the chiral point-dipole is directed either to the left or to the right, depending on its chirality. In contrast, for the case without the nanoantenna, the radiation does not show preferred directionality (shown in Figure S2, Supporting Information File 1). Importantly, the directionality could be effectively switched by changing the sign of the phase difference (circular polarization state of the point dipole).

Figure 3c,d compares the polar plots of the total (*P*_0_) and azimuthal power component (*P*_θ_) distributions. It is clear that the azimuthal power component is the dominant contribution to the radiation. This confirms that the directional emission is indeed a result of the interference between the electric dipole mode from the short gold bar and the electric quadrupole mode from the long gold bar. To quantify the observed directionality, we define the front-to-back ratio (F/B) as ratio between the total power emitted in the forward half-space to the power emitted in the backward half-space. The F/B value is 4.7 dB for the total power, and 6.0 dB for the azimuthal power component, which could be distinguished by adding a polarizer in the detection arm of a possible experiment. The directionality for the total radiation is slightly weaker than for the azimuthal component because of contributions from higher order modes excited in the long bar. Since the valley polarization of the monolayer TMDCs (corresponding to the chirality of the point-dipole emitters) depends on the polarization states of the pumping laser, we could easily tune the emission directionality from our proposed TMDC nanoantenna system just by changing the pumping polarization from left to right circular states.

As an important step of our study, we evaluate the emission enhancements brought by the plasmonic nanoantenna. The radiation enhancement is defined as the total power radiated by the two orthogonal dipole emitters (defining the chiral point dipole) coupled to the antenna, normalized to the case with no antenna. We find that the radiation is dramatically enhanced, up to 15 times at the resonant wavelength, when the chiral emitter is positioned in the center, between the two bars. In addition, we test the robustness of our design with respect to the emission wavelength. We find that the directionality is preserved in the spectral range from 680 nm to 750 nm. This broadband response makes our design suitable to control the emission from monolayer TMDCs, such as WSe_2_ in its entire emission range. Thus, the simple nanoantenna we propose here could effectively enhance the emission intensity and simultaneously tune the valley-based emission directionality from the TMDCs.

**Figure 4:**
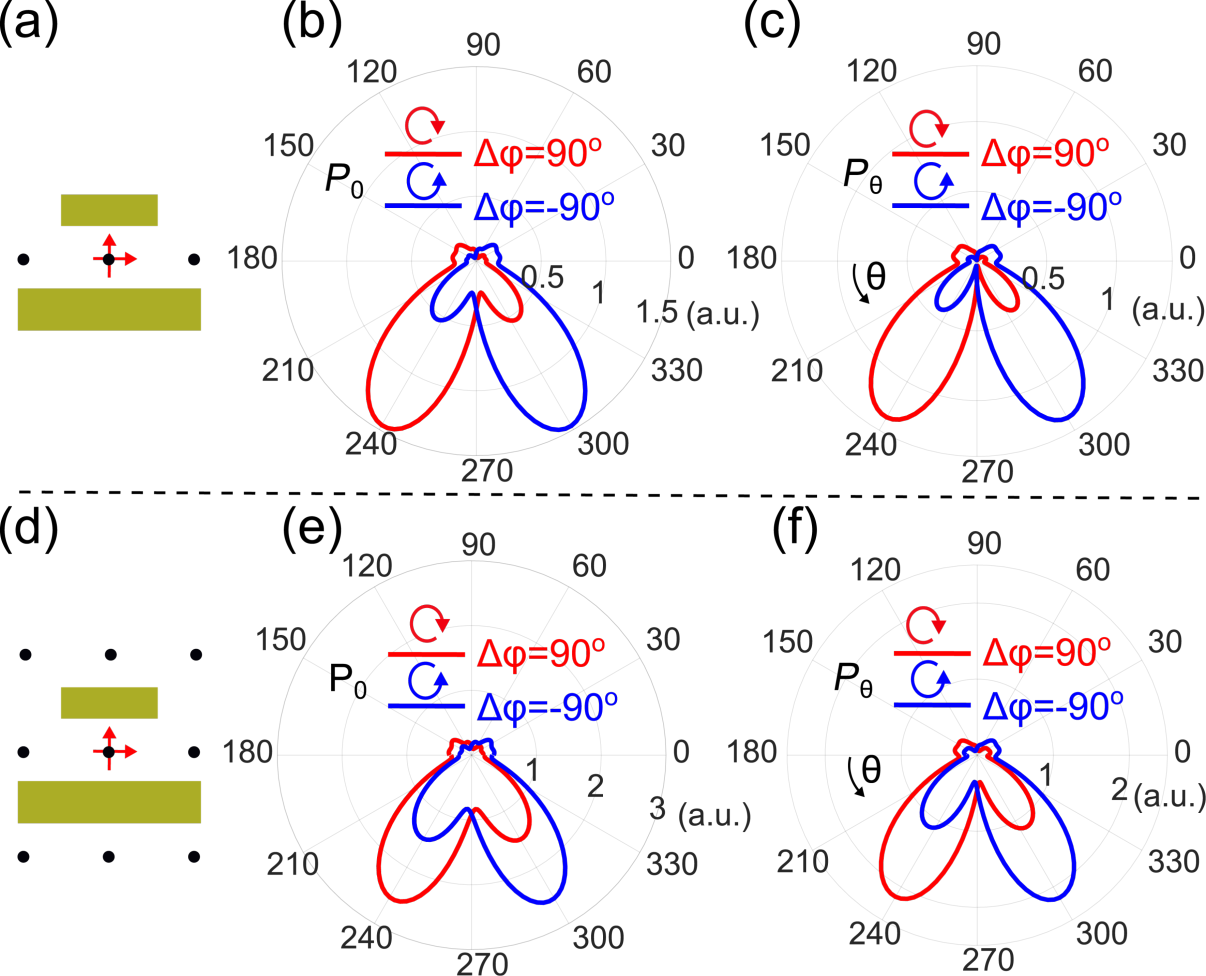
Emission pattern averaged over multiple emitters. (a) Schematic of the three emitter positions inside the gap between the two bars, used to evaluate the average effects. (b, c) Polar plot of the total (*P*_0_) and azimuthal component (*P*_θ_) of the far-field power distribution, after averaging over all three positions. (d) Schematic of the nine emitter positions used in simulations. (e, f) Polar plot of the total (*P*_0_) and the azimuthal (*P*_θ_) components of the far-field power distribution, after averaging over all the nine positions.

As discussed, the modes excited in the plasmonic nanoantenna depend on the position of the emitter. In a monolayer TMDC, however, the emitters (e.g., excitons) can be distributed randomly and homogeneously over the entire monolayer. Hence, to evaluate the directionality of emission from the average distribution of emitters on top of the double-bar antenna, we perform calculations for different positions of the point-dipole emitters and average the radiation patterns taking into account the contribution from the multiple emitters. Two cases have been considered. Firstly, we investigate the average directionality when the emitters are positioned inside the gap between the two gold bars. We calculate the emission for three different positions inside the gap, as shown in Figure 4a and then add up the radiated powers. The polar plot of the total power and the azimuthal power components are shown in Figure 4b and Figure 4c, respectively. The plots show radiation patterns, which are similar to the radiation from the central dipole position (Figure 3c,d). However, the front-to-back ratio decreases a bit after the averaging. This is due to the fact that for the locations away from the center, the directionality is reduced as compared to the central position. Nevertheless, the average directionality still remain relatively high, namely F/B = 3.4 dB for the total power and 4.2 dB for the azimuthal power component.

After averaging over more positions outside the gaps (we take nine typical positions, as shown in Figure 4d), the directionality is reduced a little further. However, as shown in Figure 4e and Figure 4f, we still observe reasonably good directionality. Thus, we can conclude, that despite the different positions of the emitters (being on both sides of the antenna) the directionality of emission is generally preserved and it can be tuned by changing the chirality of the emitters (the relative phase shift between the *X*-oriented and *Y*-oriented point-dipole emitters). Therefore, the scheme we proposed here can be used effectively to direct emission from different valleys in monolayer TMDCs (corresponding to different chiral emitters) into different directions.

We believe that with the development of material fabrication techniques, nanostructures of TMDCs [49–51] could also be readily fabricated, so one can control the position and size of the TMDCs nanosheets with respect to the antenna. Thus, a system consisting of a double-bar antenna and a TMDC nanosheet only inside the gap between the bars could in principle serve as efficient light source with tunable emission directions.

To further understand our system and seek for possible ways to improve the average directionality, we compare the total radiation power for the emitters at different positions, shown in Figure 4d. The chiral emitter placed at the central position, results in best directionality and emits two to five times stronger than the dipoles at other positions. The average directionality could be better if the central position had a much stronger emission power. Indeed, directional emission from local emitters enabled by plasmonic nanoantenna is highly sensitive to the position of the emitters [33,37]. The hotspots of the nanoantenna presented here might affect the observed directionality too, while the accurate effects rely on experimental studies. To improve the directionality, we do require antenna structures with very strong hot spots, such that the emission shows good directionality when the emitters are located at these spots. This might direct us to further improve our structures by introducing antenna shapes like bow-ties [52] or split-ring-resonators [37]. However, such structures have more geometric parameters and more higher-order modes when excited by point-dipole emitters, hence the process to optimize the geometry is difficult and time-consuming. Moreover, any complex plasmonic structures requires more demanding fabrication efforts in practice. In contrast, the proposed double-bar antenna has simple mode profiles, it is easy to optimize in size and the fabrication process is relatively straightforward.

## Conclusion

In conclusion, we propose and numerically demonstrate a novel concept to control the emission intensity and direction from different valleys in monolayer TMDCs using multi-mode plasmonic nanoantennas. We design a nanoantenna based on two gold bars, of which the dipole and quadrupole modes can be excited dominantly at the same frequency. The interference between the dipole and quadrupole modes results in directional emission, where the directionality of emission depends on the phase difference between these two modes. By emulating emission from different valleys in TMDCs with chiral point-dipole emitters of opposite chirality, we have shown that a simple two-bar plasmonic nanoantenna represents a feasible platform to direct the emission (left circular or right circular) from distinct valleys into different directions. This is due to the coupling of the chiral emitters to the two dominant plasmonic modes in the double-bar nanoantenna. Directionality of up to 6 dB and a radiation power enhancement of up to 15 times could be achieved in this coupled system. Since the valleys in TMDCs can be addressed optically, we can change the emission direction of this TMDC–nanoantenna system by simply changing the circular polarization states of the pumping light. In addition, we discuss the reasons for the reduced directionality when averaging over different positions of the emitter and propose the possible ways to address this issue, either by structuring the TMDC materials or by designing new plasmonic nanoantennas. The scheme we propose here could be potentially useful in future valley-based devices, and could in general, be applicable for circular dichroism measurements of chiral molecules.

## Supporting Information

File 1 Additional experimental data.
Supporting Information features vectorial near-field profiles for the dipole-nanoantenna system, and the radiation pattern of chiral emitters without nanoantenna.
