# Supplementary material for: Valley-selective directional emission from a transition-metal dichalcogenide monolayer mediated by a plasmonic nanoantenna
Source: Beilstein J Nanotechnol. 2018 Mar 2;9:780–8. doi: 10.3762/bjnano.9.71 (PMC5852623; doi:10.3762/bjnano.9.71)
Supplement: File 1 — Additional experimental data. Supporting Information features vectorial near-field profiles for the dipole-nanoantenna system, and the radiation pattern of chiral emitters without nanoantenna. [file Beilstein_J_Nanotechnol-09-780-s001.pdf]

Supporting Information

for

Valley-selective directional emission from a transition-metal di-chalcogenide monolayer mediated by a plasmonic nanoantenna

Haitao Chen[1,2], Mingkai Liu[1], Lei Xu[1] and Dragomir N. Neshev*[1]

Address: [1]Nonlinear Physics Centre, Research School of Physics and Engineering, Australian National University, Canberra, ACT 2601, Australia and [2]College of Advanced Interdisciplinary Studies, National University of Defense Technology, Changsha 410073, China

Email: Dragomir N. Neshev - Dragomir.Neshev@anu.edu.au

* Corresponding author

Additional computational data

Vectorial near-field profiles for the dipole–nanoantenna system

The vectorial near-field profiles when the dipole couples with the nanoantenna are shown in Figure S1. Part (a) shows the field projected on the XY-plane exited by Y-orientated dipole for the long bar, which shows a typical quadrupole profile, as the electric field vectors oscillate in two opposite directions. In part (b), we show the near-field profiles of the field excited by X-orientated dipole where we can observe typical dipole profile as all electric field vectors oscillate in the same direction. Part (c) shows the case of chiral emitter (two orthogonal dipoles with $\pi/2$ phase shift) coupling to the two-bar system, we see that quadrupole mode is predominantly excited for the long bar and the dipole mode predominantly for the short bar.

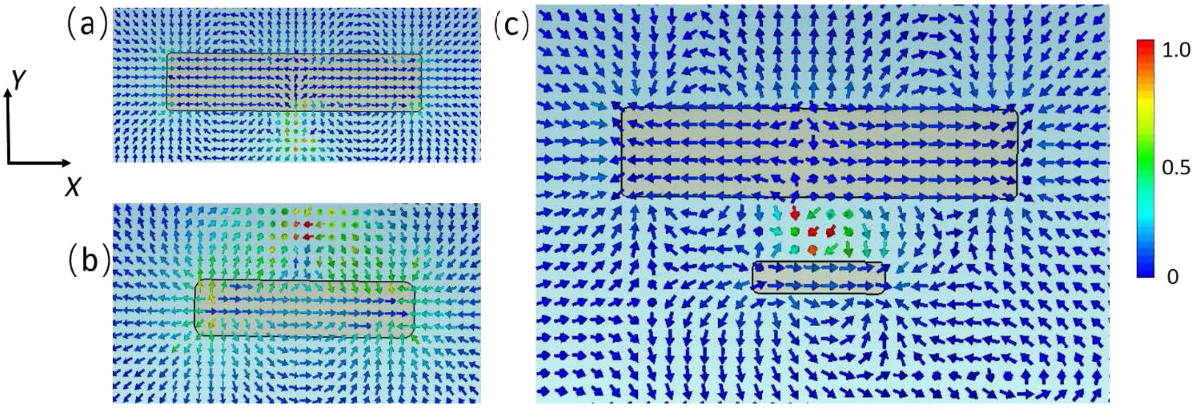

Figure S1: Top view of the vectorial electrical field near the two-bar antenna projected onto the XY-plane, (a) shows the electrical field excited by the dipole along the Y-direction for the long bar, (b) shows the electric field excited by the dipole along the X-direction for the short bar, (c) shows the coupled system consisting of the long and short bars excited by a chiral emitter.

Radiation pattern of chiral emitters without nanoantenna

The radiation patterns of chiral emitters (modelled as two orthogonal emitters with $\pm\pi/2$ phase shifts) are shown in Figure S2. Part (a) shows the radiation pattern with the dipoles sitting on top of SiO_2 substrate, part (b) shows the case without substrate. In both situations, the radiation patterns for $+\pi/2$ and $-\pi/2$ are quite similar, which is different from the cases in which coupling to the nanoantenna occurs.

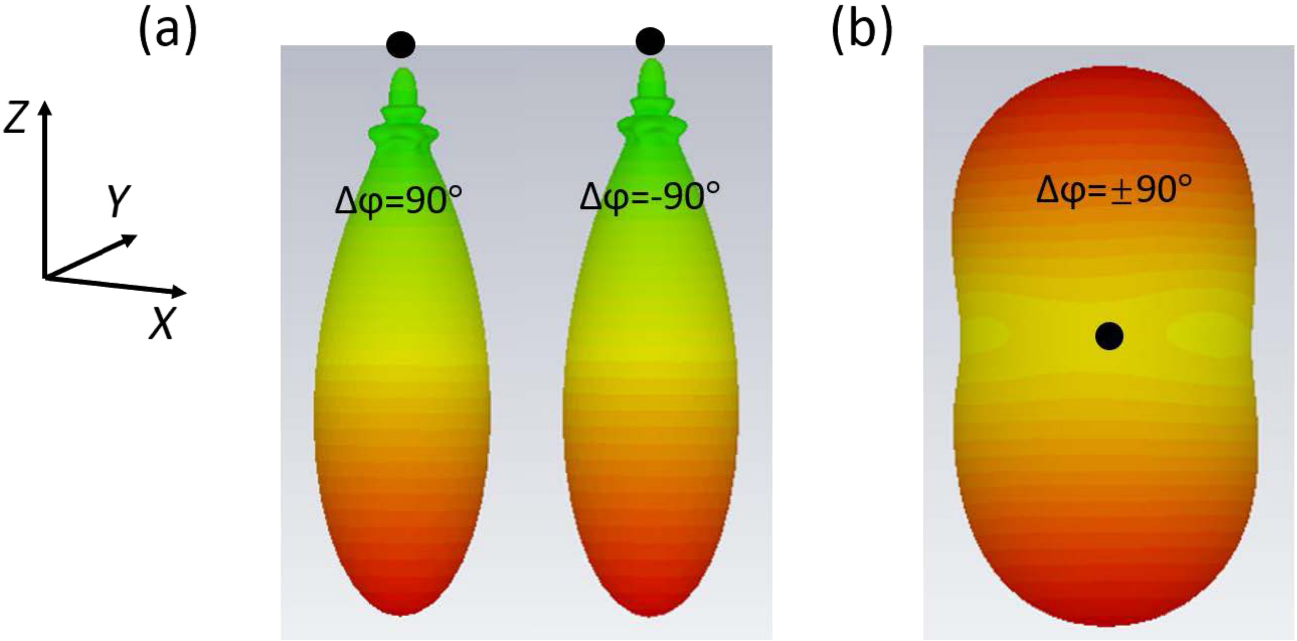

Figure S2: (a, b) Far-field radiation patterns of chiral emitter (two orthogonal dipole emitters along the X- and the Y-direction with $\pi/2$ phase shifts) with substrate (a) and without substrate (b). The black dots show the positions of the emitters.
